# SSMSNet: Scribble-Supervised Myocardial Scar Segmentation in Late Gadolinium Enhancement Images

**DOI:** 10.3390/diagnostics16121895

**Published:** 2026-06-18

**Authors:** Xuewen Liao, Kangwen Yang, Xingtao Lin, Lin Pan, Yazhou Lin, Mingjing Yang, Jiancheng Zhang

**Affiliations:** 1 Shengli Clinical Medical College of Fujian Medical University, Fuzhou 350013, China; xuewenliao@fzu.edu.cn (X.L.); asialin@fzu.edu.cn (Y.L.); 2 Department of Cardiology, Shengli Clinical Medical College of Fujian Medical University, Fuzhou University Affiliated Provincial Hospital, Fujian Provincial Hospital, No. 134 East Street, Gulou District, Fuzhou 350001, China; 3 College of Physics and Information Engineering, Fuzhou University, Fuzhou 350116, China; 231120114@fzu.edu.cn (K.Y.); 231110040@fzu.edu.cn (X.L.); panlin@fzu.edu.cn (L.P.)

**Keywords:** weakly supervised learning, cardiac anatomy segmentation, scar segmentation, local distance prior

## Abstract

**Background**: Myocardial scar segmentation from late gadolinium enhancement (LGE) cardiac magnetic resonance (CMR) images plays an important role in cardiac disease assessment and prognosis evaluation. However, accurate scar annotation is labor-intensive and requires substantial clinical expertise because scar regions are typically small, irregularly shaped, and characterized by ambiguous boundaries. Although scribble supervision provides a more practical alternative to dense annotation by substantially reducing labeling costs, the extreme sparsity of scribbles and the high similarity between scar tissue and surrounding myocardium make accurate weakly supervised segmentation challenging. **Methods**: To address these challenges, we propose SSMSNet, a novel scribble-supervised framework for myocardial scar segmentation. Specifically, a weakly supervised anatomical segmentation network is first employed to provide reliable myocardial structural priors and suppress irrelevant background interference. Subsequently, a local distance prior map is dynamically generated from scribble annotations, and a corresponding loss is introduced to enhance structural awareness and improve training stability. Meanwhile, by leveraging the spatial correlation between the myocardium and scar regions, teacher–student consistency supervision progressively recovers more complete scar structures from sparse annotations. Furthermore, a detail-aware feature enhancement module strengthens low-level representations through contextual interactions and attention mechanisms, improving the perception of scars with ambiguous boundaries. **Results**: Extensive experiments conducted on two public cardiac pathology datasets demonstrate that the proposed framework consistently outperforms state-of-the-art scribble-supervised methods and achieves competitive performance compared with fully supervised methods. **Conclusions**: The proposed SSMSNet effectively alleviates the limitations imposed by scribble annotations by integrating anatomical guidance, local distance priors, and consistency learning. These findings suggest that the framework provides an effective and annotation-efficient solution for myocardial scar segmentation in LGE CMR images.

## 1. Introduction

Myocardial scar segmentation is of substantial clinical importance for the assessment of both ischemic and non-ischemic heart diseases. The extent and spatial distribution of myocardial scar are closely associated with myocardial infarction, ventricular remodeling, and arrhythmic risk, and therefore provide important information for diagnosis, treatment planning, and prognosis evaluation [1,2]. Among current cardiac imaging modalities, late gadolinium enhancement cardiac magnetic resonance (LGE CMR) is widely regarded as the clinical reference standard for visualizing myocardial fibrosis and scar, because it enables direct depiction of damaged myocardium with high tissue contrast [3].

With the rapid development of deep learning, automatic myocardial scar segmentation on LGE CMR has achieved notable progress. Existing studies have shown that myocardial scar segmentation is highly dependent on anatomical context [4]. Therefore, many methods improve performance by explicitly exploiting the spatial relationship between the scar and the myocardium. Typical strategies include constraining scar prediction within the myocardial region, adopting cascaded or joint frameworks for myocardium and scar segmentation, and incorporating multi-plane or multi-sequence information to enhance structural consistency and reduce background interference [5,6,7,8]. These approaches have demonstrated the importance of anatomical guidance for scar delineation, especially in the presence of ambiguous boundaries, limited contrast, and complex surrounding tissues. However, most of them are developed under fully supervised settings and rely heavily on dense pixel-level annotations, which substantially limit their practical applicability in clinical scenarios.

**Figure 1:**
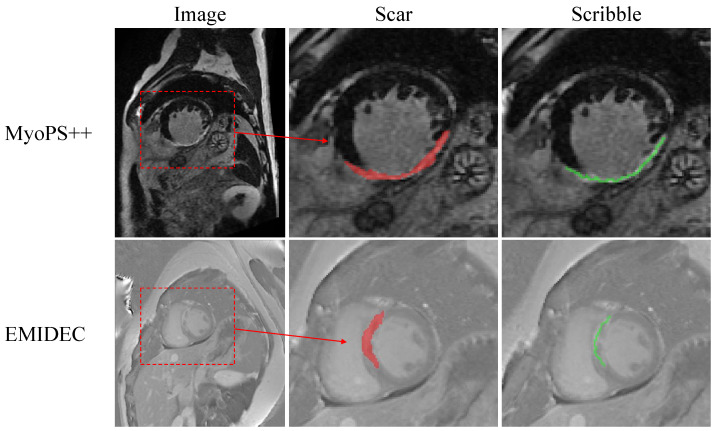
Visualization of left ventricular myocardial scar in LGE images. From left to right: the LGE image, the corresponding scar region (red), and the scribble annotations (green) used for weak supervision. The region within the red dashed box is enlarged for better visualization.

Compared with dense annotation, weak supervision offers a more practical alternative for reducing annotation burden, where only partial or sparse labels are required instead of full pixel-wise annotations. By leveraging such incomplete labels, weakly supervised methods aim to approximate the performance of fully supervised models while significantly reducing manual labeling effort and time. Among different forms of weak annotation, scribbles are particularly attractive because they require only sparse user interaction while still preserving class-specific localization cues. As illustrated in Figure 1, full pixel-wise scar annotations completely delineate the scar shape and provide detailed structural information, whereas sparse scribble labels indicate the scar extent using only a few representative pixels, offering local supervision cues. This idea has motivated a series of scribble-supervised segmentation methods, from early partial-label learning frameworks to more recent adversarial regularization, pseudo-label generation, and consistency-based training strategies. Representative methods, such as ScribbleSup [9], multi-scale adversarial attention-gates [10], CycleMix [11], DMPLS [12], and ScribFormer [13], have shown that sparse scribble annotations can substantially reduce labeling cost while maintaining competitive segmentation performance in several medical imaging tasks.

However, weakly supervised myocardial scar segmentation remains particularly challenging. Unlike relatively large and well-structured anatomical regions, myocardial scar often occupies only a small portion of the myocardium and may exhibit highly heterogeneous intensity patterns. As a result, sparse scribble labels alone are often insufficient to accurately localize the full scar extent, and naively propagating scribble supervision may easily introduce false positives into the surrounding myocardium or blood pool. Existing weakly supervised cardiac scar studies remain limited. For example, Klein et al. explored weak supervision for left ventricular fibrosis segmentation using image-level labels, but their pipeline still depended on pixel-level myocardium annotations and a multi-stage training procedure [14]. More broadly, recent scribble-supervised methods are still largely designed as generic segmentation frameworks and do not explicitly address the anatomical dependency between myocardial scar and the myocardium itself.

These limitations indicate that weakly supervised myocardial scar segmentation is not merely a sparse-label learning problem, but also a task requiring effective incorporation of anatomical and spatial priors. Specifically, three challenges should be addressed. First, sparse scribbles provide only local supervision and cannot directly describe complete scar morphology. Second, the weak contrast and fuzzy boundary of scar regions make supervision propagation highly susceptible to noise. Third, scar prediction is anatomically constrained by the myocardium, yet such structural dependency is insufficiently exploited in existing weakly supervised frameworks. A reliable myocardial prior can suppress irrelevant background responses, narrow the prediction space, and improve the structural plausibility of scar segmentation. Therefore, explicitly integrating anatomical constraints into scribble-supervised learning is highly desirable for this task.

To address these challenges, we propose SSMSNet, a novel scribble-supervised framework for myocardial scar segmentation from LGE CMR images. The framework integrates local distance priors and anatomical priors within a unified two-stage pipeline. The first stage performs anatomical prior extraction using a weakly supervised anatomical structure segmentation network to delineate the myocardium, thereby providing a region of interest and structural constraint for subsequent scar segmentation. The second stage focuses on myocardial scar segmentation under scribble supervision. To alleviate the locality of sparse labels, we introduce a dynamic distance prior derived from scar scribbles to guide the network toward learning discriminative neighborhood structures around annotated regions. To further recover more complete scar regions, we incorporate a teacher–student learning scheme that enforces region-level consistency within the myocardial area. In addition, a detail-aware feature enhancement (DFE) module is incorporated to strengthen low-level representations through local–context interaction and attention mechanisms, improving the delineation of blurred boundaries and subtle scar structures.

The main contributions of this work are summarized as follows:

- We propose a novel scribble-supervised framework, SSMSNet, which integrates local distance priors and anatomical structure priors to achieve accurate myocardial scar segmentation from LGE CMR images.
- We design a local distance prior loss based on scar scribbles, which encourages the network to learn discriminative neighborhood structures around the labeled scribble regions, thereby reducing anatomically implausible false-positive predictions and improving scar segmentation accuracy under weak supervision.
- We incorporate a teacher–student learning scheme with region-level consistency supervision within the myocardial area, enabling the progressive recovery of more complete scar regions from sparse scribbles while reducing anatomically implausible predictions.
- We introduce a detail-aware feature enhancement module to strengthen low-level feature representations through local-context interaction and attention mechanisms, thereby improving the delineation of subtle scar structures and blurred boundaries.

## 2. Literature Review

### 2.1. Myocardial Pathological Segmentation

Deep-learning-based methods for myocardial pathology segmentation in LGE-CMR mainly differ in how anatomical context and pathological features are modeled. One group of methods performs scar segmentation directly from LGE images using convolutional neural networks [15,16]. Another group improves scar quantification by coupling deep networks with additional optimization procedures, such as graph-based refinement, to obtain more regular segmentation results [17]. Anatomical dependency between pathology and myocardial structures has also been incorporated through cascaded or two-stage designs, where ventricular or myocardial regions are first identified and then used to constrain pathological segmentation [18]. In addition, multimodal and multiview frameworks have been explored to exploit complementary information from different imaging modalities or anatomical planes, thereby enhancing feature representation for myocardial pathology delineation [19,20]. Although these methods have achieved promising results, they are generally developed under fully supervised settings and require dense annotations for training.

### 2.2. Weak Supervision for Medical Image Segmentation

A major challenge in weakly supervised medical image segmentation is the insufficiency of supervisory information caused by sparse annotations. To alleviate this issue, existing methods mainly improve supervision from two perspectives. One line of research enhances representation learning by introducing consistency regularization, uncertainty modeling, or structural priors, so that the network can extract more robust and semantically meaningful features from incomplete annotations. For example, Valvano et al. incorporated multi-scale adversarial attention gates to impose shape-aware constraints during training [10], while Liu et al. proposed an uncertainty-aware mean teacher framework with transformation consistency to enhance prediction robustness under scribble supervision [21]. More recent methods further enrich structural and semantic cues through superpixel-guided label expansion, contrastive regularization, and multimodal feature enhancement, such as ScribFormer and SC-Net [13,22]. Recent studies have further emphasized the importance of structural priors and anatomy-aware learning in cardiac image analysis. For instance, shape-aware adversarial learning frameworks have been introduced to strengthen anatomical consistency with sparse scribble supervision in cardiac MRI segmentation, demonstrating the benefits of integrating morphology-aware constraints into weak-supervision pipelines [23]. In addition, anatomy-aware transformer architectures have shown that explicitly incorporating anatomical structural information can improve segmentation robustness and boundary delineation in cardiac imaging tasks [24]. These studies suggest that consistency learning and structure-aware representation enhancement are effective in mitigating the information deficiency caused by sparse annotations.

Another widely adopted line of research is pseudo-label learning, which expands sparse supervision by generating auxiliary dense labels from model predictions or collaborative inference. Representative methods include DMPLS, which dynamically mixes the outputs of dual decoders to produce more reliable pseudo labels [12], CycleMix, which combines mix augmentation with cycle consistency to regularize weak supervision [11], and TriMix, which exploits diverse-model consistency under limited supervision [25]. Recent extensions have further explored richer dense pseudo-supervision through hybrid or collaborative architectures, showing that pseudo-label refinement and multi-branch cooperation can substantially improve weakly supervised segmentation performance [26]. More recently, weakly supervised cardiac substructure segmentation methods have introduced uncertainty-guided consistency learning and structural adaptation strategies to improve segmentation reliability under limited annotations, further highlighting the importance of anatomical guidance in cardiac image analysis [27]. Nevertheless, these methods are mostly developed for relatively regular anatomical structures or generic medical segmentation tasks. When applied to pathological targets, their performance often degrades because such regions usually exhibit small size, irregular morphology, ambiguous boundaries, and strong dependence on surrounding anatomical context.

## 3. Methodology

**Figure 2:**
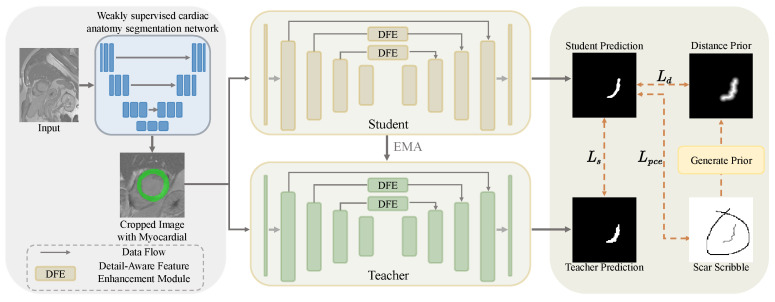
Overview of the proposed scribble-supervised scar segmentation framework (SSMSNet). A weakly supervised anatomical structure segmentation network is first applied to extract the myocardial ROI from the original image. Scar segmentation is subsequently performed on the cropped ROI using a teacher–student network with inserted detail-aware feature enhancement (DFE) modules, where the student branch is supervised by the partial cross-entropy loss and the local distance prior loss, while a structural prior consistency loss is imposed between teacher and student predictions within the myocardium region.

The overall framework of the proposed scribble-supervised myocardial scar segmentation framework, together with the associated loss functions, is illustrated in Figure 2.

### 3.1. Overall Structure

Since myocardial scar regions usually occupy only a small portion of LGE images, directly performing scribble-supervised scar segmentation on the entire image is highly susceptible to background interference. To address this issue, we adopt a two-stage strategy that decomposes the task into myocardium localization and scar segmentation.

In the first stage, a weakly supervised anatomical segmentation network is employed to segment the myocardium and generate the corresponding region of interest (ROI). By restricting subsequent analysis to the myocardial ROI, irrelevant background regions can be effectively suppressed, thereby improving both the efficiency and reliability of scar segmentation. In the second stage, myocardial scar segmentation is performed within the cropped ROI using a teacher–student framework. Specifically, a local distance prior derived from scar scribbles is introduced to guide the propagation of supervisory signals beyond the sparsely annotated pixels. Meanwhile, a structural consistency constraint within the myocardium region is imposed between the teacher and student predictions, enabling the model to progressively recover more complete scar structures from sparse supervision. In addition, a detail-aware feature enhancement (DFE) module is incorporated to strengthen low-level feature representation, thereby improving the discriminability of scar regions and the delineation accuracy of ambiguous boundaries.

### 3.2. Local Distance Prior Loss

In scribble-supervised myocardial scar segmentation, only a very limited subset of pixels is annotated, resulting in extremely sparse supervision. Under this setting, conventional weakly supervised methods that mainly rely on salient target localization are often inadequate for scar segmentation. This is because myocardial scar regions in LGE images are usually small, irregularly shaped, and characterized by ambiguous boundaries, making them far less visually distinctive than anatomical structures such as the myocardium and blood pool. As a result, the network tends to focus on these relatively salient anatomical regions while failing to accurately localize the scar. To address this issue, we introduce a local distance prior loss to encourage the network to learn a spatially coherent scar probability distribution in the neighborhood of scar scribbles. The basic intuition is that pixels closer to scar scribbles are more likely to belong to the scar region and should therefore receive stronger supervision, whereas pixels farther away should contribute less to the optimization. Unlike global distance regularization, the proposed prior is imposed only within a local neighborhood around the scribbles, thereby avoiding overly strong global shape constraints and reducing the risk of oversmoothing irregular scar structures.

Let $I\in R^{H\times W}$ denote the input image, and let $Y_{s}\in {\left\{0,1,2\right\}}^{H\times W}$ denote the corresponding scribble annotation, where 0, 1, and 2 indicate background, scar scribble, and unknown labels, respectively. We first collect all scar scribble pixels to form a foreground scribble set $S=\{\left(i,j\right)|Y_{s}\left(i,j\right)=1\}$. For an arbitrary pixel $x=(x_{1},x_{2})$ and a scribble pixel $y=(y_{1},y_{2})\in S$, we define their weighted Euclidean distance as

(1) $$d\left(x,y\right)=\sqrt{\lambda \sum_{k=1}^{2}{\left(x_{k}-y_{k}\right)}^{2}},$$

where λ>0 is a distance scaling factor that controls the decay rate of the distance prior. Based on this definition, the truncated minimum distance from pixel *x* to the scribble set is

(2) $$D\left(x\right)=\min\left(\omega ,\min_{y\in S}d\left(x,y\right)\right),$$

where ω>0 is an upper truncation threshold. Distances larger than ω are clipped, ensuring that only pixels within a local neighborhood of the scribbles contribute to the distance-prior constraint.

**Figure 3:**
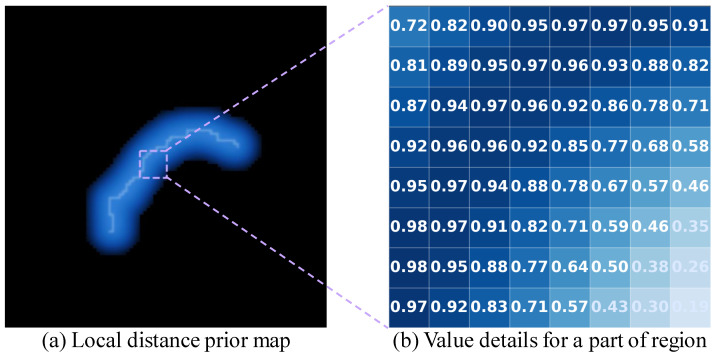
(**a**) Visualization of the local distance prior map. (**b**) Detailed values of a selected region within the map, showing a progressive decrease as the distance from the scar scribble increases.

To construct the local distance prior map, the truncated distance D(x) is inversely transformed and normalized to the range [0,1], yielding M(x). As illustrated in Figure 3, pixels closer to the scribbles are assigned larger prior values, whereas pixels farther away receive smaller values. In this way, the prior provides stronger supervision in the vicinity of annotated scar pixels while gradually suppressing the influence of distant regions. A binary local mask $\Gamma_{L}\left(x\right)$ is then obtained by thresholding M(x) at a predefined local threshold $\tau_{d}$:

(3) $$\Gamma_{L}\left(x\right)=\left\{\begin{array}{cc} 1, & M\left(x\right)>\tau_{d},\\ 0, & \mathrm{otherwise}, \end{array}\right.$$

ensuring that the distance prior is imposed only within the selected local region.

Let P(x) denote the predicted foreground probability of the scar class at pixel *x*. The local distance prior loss is defined as an $L_{1}$ loss over the masked region:

(4) $$L_{d}=\frac{\sum_{x}\Gamma_{L}\left(x\right)\;\left|P(x)-M(x)\right|}{\sum_{x}\Gamma_{L}\left(x\right)+\epsilon },$$

where ϵ is a small constant to avoid division by zero. By enforcing consistency between the network prediction and the local distance prior within the masked region, this loss enables sparse scribble supervision to propagate in a controlled manner around annotated scar pixels, focusing the network on scar-related local structures while preventing excessive expansion and structural oversmoothing.

### 3.3. Structural Prior Consistency Loss

To obtain more stable and reliable supervision under scribble-based learning, we adopt a teacher–student framework in the myocardial scar segmentation stage. The teacher network is updated from the student network using an exponential moving average (EMA), which generally produces smoother and more stable predictions than the student network. Based on the extracted anatomical prior, we further enforce structural consistency by introducing a structural prior consistency loss. Specifically, the proposed loss consists of a region-level consistency term and a high-confidence pseudo-label supervision term. The former enforces structural consistency between the student and teacher predictions within the myocardium, while the latter applies stronger supervision only to highly confident teacher predictions, thereby reducing the influence of unreliable pseudo-labels.

Let $P_{s}\left(x\right)$ and $P_{t}\left(x\right)$ denote the predicted foreground probabilities of the student and teacher networks at pixel *x*, respectively. Let R(x)∈{0,1} denote the myocardial mask, where R(x)=1 indicates that pixel *x* lies inside the myocardium. To encourage the student and teacher networks to produce structurally consistent predictions within the myocardial region, we define the region-level consistency loss as

(5) $$L_{sd}=1-\frac{2\sum_{x}R\left(x\right)\;P_{s}\left(x\right)P_{t}\left(x\right)}{\sum_{x}R\left(x\right)\;P_{s}\left(x\right)+\sum_{x}R\left(x\right)\;P_{t}\left(x\right)+\epsilon }$$

where ϵ is a small constant to avoid division by zero. This term maximizes the overlap between student and teacher predictions inside the myocardium, thereby promoting structurally coherent scar estimation under anatomical constraints.

Although the teacher network provides relatively stable pseudo-labels, its predictions in low-confidence regions may still contain considerable noise. Directly enforcing consistency on all pixels may therefore impair optimization stability and segmentation accuracy. To alleviate this issue, we further impose supervision only on highly confident teacher predictions. Specifically, a confidence mask is constructed as

(6) $$C\left(x\right)=\left\{\begin{array}{cc} 1, & P_{t}\left(x\right)>\tau_{p}\;\mathrm{or}\;P_{t}\left(x\right)<1-\tau_{p},\\ 0, & \mathrm{otherwise} \end{array}\right.$$

where $\tau_{p}$ denotes the confidence threshold of the teacher prediction. Based on this mask, the high-confidence pseudo-label supervision loss is defined as

(7) $$L_{sc}=\frac{\sum_{x}C\left(x\right)R\left(x\right){\left(P_{s}\left(x\right)-P_{t}\left(x\right)\right)}^{2}}{\sum_{x}C\left(x\right)R\left(x\right)+\epsilon }$$

The above strategy enables the model to selectively exploit reliable pseudo-label regions while avoiding the introduction of noisy supervision from uncertain areas. Consequently, the training stability of the student network and the accuracy of scar prediction can be further improved. By combining anatomical constraint with confidence-aware teacher guidance, the final structural prior consistency loss is formulated as

(8) $$L_{s}=L_{sd}+L_{sc}$$

### 3.4. Detail-Aware Feature Enhancement Module

**Figure 4:**
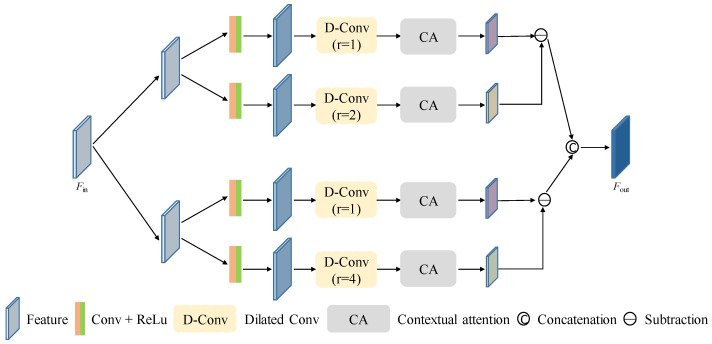
Detail-aware feature enhancement module (DFE).

In LGE MRI images, scar regions often exhibit grayscale intensity and textural characteristics that are highly similar to those of the surrounding myocardium. This similarity, particularly at the boundaries where transitions are indistinct and intensity contrast is weak, makes it challenging for the network to effectively identify and extract scar-related features, thereby compromising segmentation performance. To address this issue, we propose a detail-aware feature enhancement (DFE) module, designed to emphasize subtle differences between scar tissue and adjacent structures at low-level feature stages. As shown in Figure 4, this module constructs two parallel feature-extraction branches to capture fine-grained local and broader contextual information, respectively. By computing the differences between local and contextual features, the module guides the network to focus on potential scar regions that may be imperceptible in local areas but exhibit distinguishable patterns at a larger spatial scale. Compared with directly using low-level features, this approach enhances the subtle structural variations along the scar boundaries, suppresses background interference, and preserves high-resolution fine details.

Specifically, given an input low-level feature map $F_{in}\in R^{C\times H\times W}$, the proposed DFE module first distributes $F_{in}$ into two parallel contrastive branches to model feature discrepancies under different receptive field settings. In each branch, the input features are further fed into two subpaths. Each subpath starts with a convolutional layer followed by a ReLU activation to obtain an initial low-level representation, which is then processed by a dilated convolution with a specific dilation rate to capture structural responses at a corresponding spatial scale. After that, a contextual attention (CA) unit, inspired by the pixel-wise contextual attention mechanism in PiCANet [28], is employed to adaptively reweight contextual feature responses for each spatial location, such that informative regions can be emphasized while irrelevant background interference is suppressed. The outputs of the two subpaths within each branch are then subtracted to generate a contrast-enhanced feature map, which highlights subtle structural variations that are difficult to capture using only local receptive fields. Finally, the contrastive outputs from the two branches are concatenated along the channel dimension to form the enhanced feature representation $F_{out}$. Through this dual-branch design, the DFE module explicitly captures the discrepancy between local details and multi-scale contextual cues, thereby strengthening boundary-sensitive representations and improving the discriminability of myocardial scar regions with small size, weak contrast, and ambiguous boundaries.

### 3.5. Loss Function

Based on the above designs, the training objective of the proposed myocardial scar segmentation framework consists of three components: the partial cross-entropy loss, the local distance prior loss, and the structural prior consistency loss. The overall loss function is formulated as

(9) $$L=L_{pce}+\lambda_{d}L_{d}+\lambda_{s}L_{s}$$

where $\lambda_{d}$ and $\lambda_{s}$ are weighting coefficients used to balance the contributions of the local distance prior loss and the structural prior consistency loss, respectively.

## 4. Experiments and Results

### 4.1. Datasets and Preprocessing

To validate the effectiveness of the proposed framework for weakly supervised myocardial scar segmentation, experiments were conducted on two publicly available cardiac pathology datasets: the CARE 2024 Myocardial Pathology Segmentation (MyoPS++) dataset and the EMIDEC Challenge dataset.

The MyoPS++ dataset contains 181 LGE CMR images, with manual annotations for the left ventricle (LV), LV scar, and LV edema. Following a random split, 145 cases were used for training, and the remaining 36 cases were reserved for testing. The EMIDEC dataset consists of 100 cases, including 67 pathological cases and 33 normal cases. Since this study focuses on myocardial pathology segmentation, only the 67 pathological cases were included in the experiments. Among them, 54 cases were randomly selected for training, while the remaining 13 cases were used for testing.

Since the proposed framework consists of anatomical structure segmentation and myocardial scar segmentation, different annotation preparation and preprocessing procedures were adopted for the two tasks. For anatomical structure segmentation, scribble annotations of cardiac anatomical structures were manually annotated by experienced clinicians and further reviewed by an additional observer for quality control. The annotation protocol followed the publicly available cardiac anatomical scribble annotation strategy, where sparse strokes were used to indicate representative anatomical regions while leaving the remaining areas unlabeled. Before training, all images were resampled to a uniform in-plane resolution of 1×1 mm^2^ and normalized to the range of [0,1]. To reduce irrelevant background information and focus on cardiac structures, images were cropped around the heart region and resized to 256×256 pixels before being used for anatomical structure segmentation. For myocardial scar segmentation, sparse scar scribble annotations were generated from dense scar masks manually delineated by experienced clinicians. Specifically, dense annotations were converted into sparse scribbles to simulate weak supervision while maintaining representative spatial characteristics of scar regions. For scar segmentation preprocessing, images were further cropped according to the predicted myocardial structures obtained from anatomical segmentation to extract myocardium-centered regions. The cropped images were subsequently resized to 128×128 pixels and used as inputs for myocardial scar segmentation.

### 4.2. Implementation Details

#### 4.2.1. Anatomical Structure Segmentation Configuration

For anatomical structure segmentation, the network was implemented using DMPLS [12]. This network was implemented in PyTorch 1.11.0 and trained on an NVIDIA GeForce RTX 4090 GPU. During training, data augmentation strategies were applied to improve robustness and reduce overfitting, including random rotation, random flipping, and random noise perturbation. The network parameters were optimized using stochastic gradient descent (SGD) with a momentum of 0.9 and a weight decay of $1\times 10^{-4}$. The poly learning rate strategy was adopted to dynamically adjust the learning rate during training. The batch size, total training iterations, and weighting coefficient λ were set to 12, 60k, and 0.5, respectively. During inference, predictions were generated slice-by-slice and subsequently stacked into three-dimensional volumes for evaluation.

#### 4.2.2. Myocardial Scar Segmentation Configuration

The myocardial scar segmentation network was implemented using PyTorch 1.11.0 and trained on an NVIDIA GeForce RTX 4090 GPU. To improve model robustness and reduce overfitting, data augmentation was employed during training. Specifically, random rotation and random rotation-flipping transformations were applied to training samples while maintaining spatial consistency among images, scribble labels, local distance maps, and myocardial masks. Network optimization was performed using stochastic gradient descent (SGD) with a momentum of 0.9 and a weight decay of $1\times 10^{-4}$. The batch size was set to 16, and the maximum number of training iterations was set to 80k. For local distance prior loss, the range coefficient λ was set to 0.7 and the threshold parameter $\tau_{d}$ was set to 0.9. The local distance weight $\lambda_{d}$ was dynamically adjusted during training, with an initial value of 1.0 that gradually decreased to 0.1 after the warm-up stage. For structural prior consistency loss, the confidence threshold $\tau_{p}$ for generating reliable pseudo labels was set to 0.8. The maximum pseudo-supervision weight ω was set to 0.5. All experiments were conducted using a fixed random seed of 2022 to ensure reproducibility. The consistency weighting coefficient $\lambda_{s}$ was dynamically increased after the warm-up stage, where its value gradually increased from 0 to a maximum value of 0.5.

The hyperparameters in the proposed framework were selected through a combination of empirical initialization and validation-based refinement. Unless otherwise specified, identical hyperparameter settings were used for both the MyoPS++ and EMIDEC datasets. This design was adopted to evaluate the generalization ability of the proposed framework across datasets with different imaging characteristics and annotation distributions, while avoiding dataset-specific parameter tuning.

### 4.3. Results on the MyoPS++ Dataset

To validate the effectiveness and competitiveness of the proposed method, we first compared the proposed method with three representative weakly supervised segmentation methods under scribble supervision, including USTM [21], DMPLS [12], and ScribFormer [13]. To further evaluate the gap between weakly supervised learning and fully supervised performance, we also included three widely used fully supervised segmentation methods, including U-Net [29], UNet++ [30], and nn-UNet [31].

It should be noted that the fully supervised methods were trained using dense myocardial scar annotations rather than sparse scribble labels. Therefore, these methods were not intended as directly comparable weakly supervised baselines, but rather served as reference models to indicate the achievable upper-bound segmentation performance under full supervision. For fair comparison, all methods were implemented and trained under identical experimental settings, including the same training/testing splits, preprocessing procedures, and evaluation protocols. Hyperparameters for competing methods were selected according to their original implementations or official recommendations and further tuned within comparable ranges to achieve stable performance. Segmentation performance was quantitatively evaluated using the Dice similarity coefficient (DSC), 95% Hausdorff distance (HD95), and average surface distance (ASD).

As reported in Table 1, the proposed SSMSNet achieves the best performance among all weakly supervised methods, with a DSC of 52.7%, an HD95 of 13.827 mm, and an ASD of 4.542 mm. Compared with USTM, DMPLS, and ScribFormer, our method yields consistent improvements across all three metrics, indicating superior overlap accuracy and boundary delineation. In particular, the notable reductions in HD95 and ASD suggest that the proposed method produces more spatially consistent scar contours and more accurate boundary localization under sparse supervision.

Although the fully supervised methods still achieve slightly better overall performance, SSMSNet approaches the level of some supervised baselines and even outperforms U-Net++ in DSC. This result demonstrates that the proposed framework can effectively exploit sparse scribble annotations to recover meaningful scar structures. We attribute this advantage to the joint effect of the local distance prior and the myocardium-constrained structural consistency learning, which together enhance local scar awareness and improve structural coherence during optimization.

**Figure 5:**
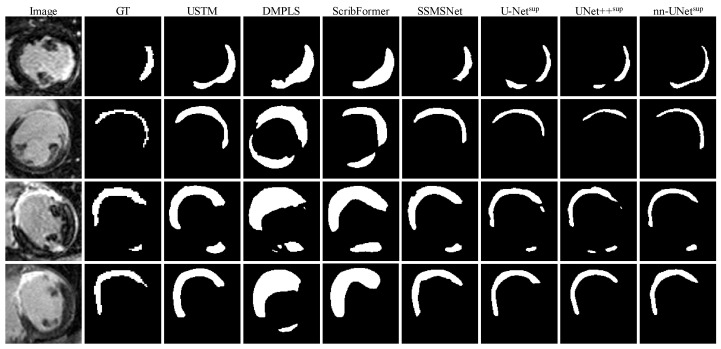
Visualization of different scar segmentation methods on the MyoPS++ dataset.

Figure 5 further presents qualitative comparisons of several representative cases. Fully supervised methods, especially nn-UNet, generally provide more complete scar segmentation, which is consistent with the quantitative results in Table 1. In contrast, conventional weakly supervised methods often fail to accurately localize small or irregular scar regions and tend to produce incomplete or fragmented predictions. Benefiting from the proposed supervision strategy, SSMSNet is able to better delineate scar regions and preserve their structural integrity, further verifying its effectiveness on the MyoPS++ dataset.

### 4.4. Results on the EMIDEC Dataset

We further evaluated the proposed method on the EMIDEC dataset and compared it with three representative scribble-supervised segmentation methods, including USTM, DMPLS, and ScribFormer, as well as three widely used fully supervised baselines, i.e., U-Net, UNet++, and nn-UNet.

As reported in Table 2, the proposed SSMSNet achieves the best overall performance among all weakly supervised methods, with a DSC of 49.2%, an HD95 of 11.247 mm, and an ASD of 4.809 mm. Compared with USTM, DMPLS, and ScribFormer, our method yields substantial improvements across all three evaluation metrics, indicating that it can more accurately localize myocardial scar regions and produce more spatially consistent segmentation boundaries under sparse scribble supervision. In particular, the large reductions in HD95 and ASD demonstrate the superiority of the proposed method in boundary delineation and contour regularity, which is especially important for scar regions with irregular shapes and ambiguous appearances.

Notably, SSMSNet also shows highly competitive performance compared with the fully supervised baselines. Although nn-UNet still achieves the highest DSC and the best ASD, the proposed method outperforms both U-Net and UNet++ in DSC and HD95, and also achieves better ASD than these two supervised baselines. These results suggest that the proposed framework is capable of effectively exploiting sparse scribble annotations to learn discriminative scar representations, even approaching the performance of fully supervised methods on this challenging dataset.

**Figure 6:**
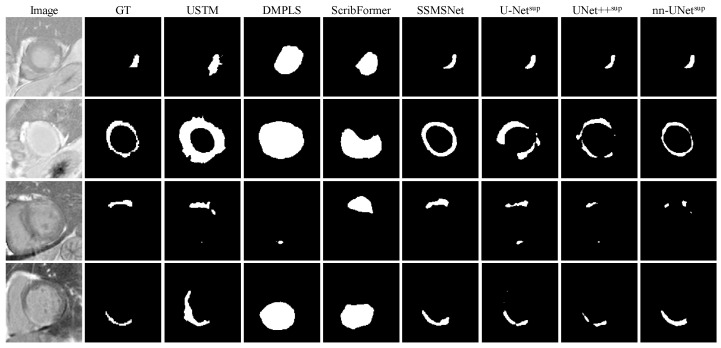
Visualization of different scar segmentation methods on the EMIDEC dataset.

Figure 6 presents qualitative comparisons on several representative cases from the EMIDEC dataset. As can be observed, conventional weakly supervised methods often fail to accurately capture the shape and extent of scar regions, and tend to produce over-smoothed, incomplete, or severely biased predictions. In contrast, the proposed SSMSNet is able to generate scar segmentation results that are much closer to the ground truth, with improved localization accuracy and better preservation of structural details. In several cases, its predictions are even visually comparable to those produced by fully supervised methods. These qualitative observations are consistent with the quantitative results in Table 2, further confirming the effectiveness and robustness of the proposed method on the EMIDEC dataset.

### 4.5. Failure Case Discussion

**Figure 7:**
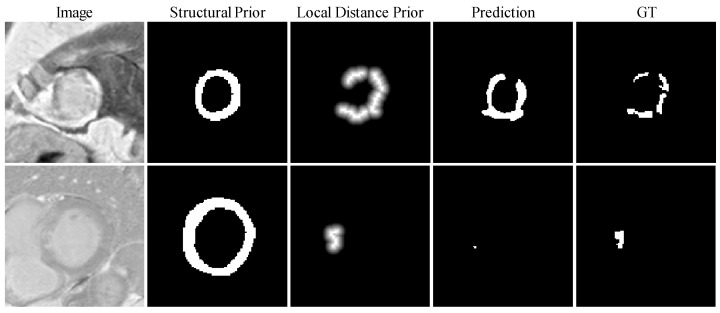
Representative challenging cases for myocardial scar segmentation. Columns correspond to the input image, structural prior, local distance prior, predicted segmentation, and ground truth, respectively.

Although the proposed framework achieves competitive performance across two datasets, several challenging cases still lead to incomplete scar delineation. Representative examples are illustrated in Figure 7, where structural priors, local distance priors, and final segmentation results are visualized simultaneously to facilitate interpretation.

In the first case, the scar region exhibits fragmented and spatially discontinuous morphology with ambiguous boundaries and multiple separated components. Although the structural prior successfully constrains the prediction within anatomically plausible myocardial regions and the local distance prior provides localized supervision around sparse annotations, the predicted segmentation still fails to accurately recover the complete scar distribution and connectivity patterns. This observation suggests that sparse supervision remains insufficient for capturing complex spatial relationships when scar regions are highly fragmented and irregularly distributed. The second case represents a small-volume scar with weak image contrast and limited annotation information. Since the generated local distance prior is derived from sparse scribble annotations, only a relatively limited region receives strong supervision, causing the network to produce under-segmented predictions and even miss parts of the lesion. This observation indicates that very small pathological regions remain challenging under sparse supervision.

Overall, these failure cases provide further insight into the strengths and limitations of the proposed framework. The structural prior effectively constrains predictions within anatomically plausible regions, while the local distance prior guides learning toward areas surrounding sparse annotations. However, when scar morphology becomes highly fragmented, lesion size is extremely small, or appearance contrast is weak, the limited supervision provided by sparse annotations may still restrict accurate lesion delineation.

### 4.6. Sensitivity to Anatomical Prior

To evaluate the robustness of the proposed framework to inaccuracies in anatomical priors and analyze the propagation of myocardial segmentation errors to scar segmentation, we conducted a perturbation study by artificially modifying the predicted myocardial masks using morphological dilation and erosion operations. The perturbation magnitude was quantified using the average symmetric surface distance (ASSD) between the perturbed myocardial masks and the original predicted masks, allowing controlled simulation of anatomical prior inaccuracies with different severity levels.

Quantitative results are summarized in Table 3. As the perturbation magnitude increased, segmentation performance gradually deteriorated under both dilation and erosion settings, indicating that the quality of anatomical priors directly affects downstream myocardial scar segmentation performance. Nevertheless, mild-to-moderate perturbations only caused relatively limited performance degradation. For example, when the prior error increased from 0.272±0.043 mm to 1.026±0.126 mm under dilation perturbation, the DSC decreased from 0.527 to 0.501, suggesting that the proposed framework maintains reasonable robustness to imperfect anatomical priors.

A comparison between dilation and erosion reveals asymmetric sensitivity to different types of prior errors. Specifically, erosion consistently resulted in larger performance degradation than dilation, particularly under severe perturbations, where the DSC decreased to 0.487 and HD95 increased to 23.150 mm. This observation suggests that missing myocardial regions are more detrimental than introducing redundant surrounding tissues, since incomplete anatomical priors may remove critical contextual information required for accurate scar localization, whereas moderate over-segmentation mainly introduces additional non-target regions without substantially affecting segmentation accuracy.

**Figure 8:**
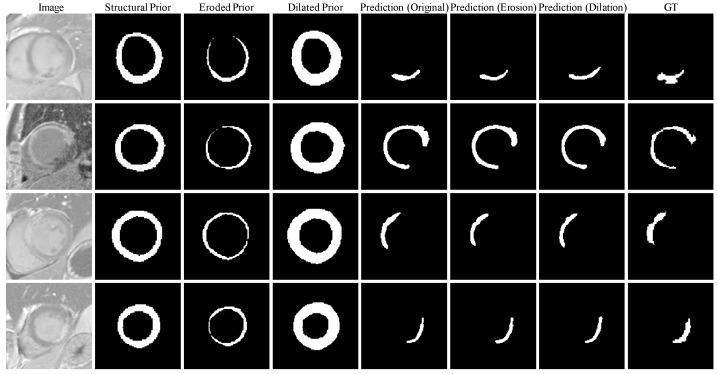
Visualization of segmentation results of myocardial structure prior under different perturbations.

Representative examples of myocardial scar segmentation under severe anatomical prior perturbations are illustrated in Figure 8. As the perturbation magnitude increases, distorted myocardial prior information gradually propagates to the downstream scar segmentation process, resulting in incomplete scar delineation. Specifically, inaccurate anatomical priors lead to noticeable deviations in scar morphology and boundary estimation, demonstrating the dependency of downstream segmentation performance on prior quality. Nevertheless, despite these perturbations, the proposed framework maintains relatively stable segmentation results even under large prior errors, further demonstrating its robustness to imperfect anatomical guidance and its ability to tolerate inaccuracies in upstream myocardial segmentation.

### 4.7. Ablation Study

To further verify the contributions of the proposed modules and loss designs to scribble-supervised myocardial scar segmentation, we conducted ablation studies on the MyoPS++ dataset.

To investigate the effect of the distance decay factor λ in the local distance prior loss, we performed a dedicated ablation study. Specifically, λ was set to $e^{5}$, $e^{6}$, $e^{7}$, and $e^{8}$, respectively, and the model was trained and evaluated under the same experimental settings. The segmentation results under different distance decay factors are reported in Table 4.

As shown in Table 4, the overall segmentation performance first improves and then slightly declines as the decay factor λ increases. The best performance is achieved at $\lambda =e^{7}$, where the Dice score reaches 52.7% and HD95 decreases to 15.286 mm. These results indicate that an appropriate distance decay factor can achieve a better balance between local supervision propagation and noise suppression, thereby effectively improving scar segmentation performance under weak supervision.

The distance threshold $\tau_{d}$ is used to control the effective range of local distance supervision, i.e., the distance prior is imposed only within the region close to the scribble annotations, to avoid introducing unreliable supervision from distant areas. To analyze the influence of this threshold, we further conducted ablation experiments with different values of $\tau_{d}$ under the same training settings. The results are summarized in Table 5.

It can be observed that different distance thresholds have a clear influence on segmentation performance. When $\tau_{d}=0.9$, the model achieves the best Dice score of 52.8%, indicating that a relatively large threshold can enlarge the effective supervision region to a certain extent. However, the corresponding HD95 is 22.458 mm, which is relatively high, suggesting that an excessively large threshold may include regions far away from the scribbles into the supervision range and thus introduce noise, leading to less accurate boundary localization.

To further analyze the influence of the confidence threshold $\tau_{p}$ in the teacher network, we performed ablation experiments with different values of $\tau_{p}$. The results are shown in Table 6.

From Table 6, it can be seen that the confidence threshold significantly affects segmentation performance. The best overall performance is obtained when $\tau_{p}=0.80$, where the Dice score reaches 52.9%, while HD95 and ASD are reduced to 15.574 mm and 7.525 mm, respectively, both outperforming the other settings. In comparison, when the threshold is too low, more pseudo-label information can be retained, but low-confidence noisy regions are also more likely to be introduced. Conversely, when the threshold is further increased, the number of high-confidence pixels participating in supervision decreases, leading to insufficient effective supervision. Overall, an appropriate confidence threshold can better balance pseudo-label reliability and supervision utilization.

To further verify the contribution of each proposed component and loss function, we performed an ablation study by progressively introducing the local distance prior loss $L_{d}$, structural prior consistency loss $L_{s}$, the detail-aware feature enhancement module (DFE), and the teacher–student (TS) learning framework on top of the baseline model. The results are summarized in Table 7.

As shown in Table 7, each component consistently improves segmentation performance. Adding $L_{d}$ alone increases the Dice score from 0.303 to 0.331, demonstrating that the local distance prior effectively enhances supervision around scar scribbles. Incorporating the DFE module or TS further boosts performance, reflecting the benefits of enhanced feature representation and consistency-guided learning. Introducing $L_{s}$ on top of $L_{d}$ and TS notably reduces HD95 and ASD, indicating improved structural coherence under myocardial constraints. The combination of all components achieves the best overall performance with a Dice score of 0.527, an HD95 of 13.828 mm, and an ASD of 4.546 mm. These results confirm that each component contributes positively and that their integration produces the most accurate and robust myocardial scar segmentation.

## 5. Discussion

This study investigates weakly supervised myocardial scar segmentation under scribble annotations. Compared with fully supervised methods, weakly supervised methods substantially reduce the annotation cost and labor requirements. However, myocardial scars are usually small, irregularly shaped, and characterized by ambiguous boundaries, making the supervision provided by sparse scribbles extremely limited. In addition, the intensity similarity between scar tissue and surrounding myocardium poses challenges for conventional weakly supervised methods that rely on appearance cues, often resulting in incomplete or anatomically implausible predictions. To address these challenges, we explicitly decompose the task into myocardium localization and scar segmentation, and integrate anatomical priors, local structural constraints, and consistency learning into a unified framework.

Anatomical priors provide coarse structural guidance by restricting scar prediction to anatomically relevant regions and suppressing irrelevant background responses. This design is motivated by the strong anatomical dependence between myocardial scars and the surrounding myocardium, as demonstrated in previous studies [4,5]. However, anatomical constraints alone cannot effectively recover detailed scar morphology under sparse supervision. Therefore, local distance priors generated from scribble annotations are introduced to strengthen supervision around sparse annotations and improve optimization stability. Specifically, the prior is constructed based on the Euclidean distance between image pixels and the annotated scribbles, enabling supervision strength to decay smoothly. Building upon these priors, structural consistency learning further propagates reliable information within myocardial regions through teacher-guided supervision, progressively recovering more complete scar structures from sparse annotations. In addition, the detail-aware feature enhancement module compensates for weak appearance cues by strengthening low-level feature representation and improving boundary perception. Together, these components provide complementary anatomical, spatial, and semantic constraints for myocardial scar segmentation under scribble supervision.

The additional sensitivity analysis further demonstrates that the proposed framework maintains relatively stable performance under moderate perturbations of anatomical priors, suggesting a certain degree of robustness to imperfect myocardial guidance. Nevertheless, the performance degradation observed under severe perturbations indicates that the framework still depends on reliable anatomical localization. Furthermore, failure case analysis reveals that highly irregular scars with discontinuous morphology and small lesions with low contrast remain challenging, primarily because sparse annotations provide insufficient contextual information for complete structural recovery.

Despite these encouraging results, several limitations remain. First, although the proposed framework exhibits robustness to moderate anatomical prior errors, severe inaccuracies in myocardium localization can still propagate to downstream scar segmentation. Second, the local distance prior relies on manually selected hyperparameters, whose optimal settings may vary under different annotation conditions or imaging characteristics. Third, the current implementation is based on slice-wise processing and does not explicitly exploit inter-slice spatial continuity, which may limit volumetric consistency. Fourth, although experiments on two public datasets demonstrate promising performance, the generalization ability of the proposed framework on larger-scale datasets with more diverse imaging characteristics still requires further validation. Fifth, while the DFE module improves feature representation through dual-branch contextual interaction and attention mechanisms, the additional architectural complexity and computational cost (e.g., inference time, parameter count, FLOPs) have not been quantitatively analyzed, leaving deployment efficiency to be further evaluated.

From a practical perspective, even moderate segmentation performance can provide clinically useful preliminary localization of myocardial scars, reducing manual annotation workload and supporting downstream analysis such as scar burden quantification or treatment planning. Therefore, despite the inherent challenges of scribble-supervised scar segmentation, the proposed framework offers an annotation-efficient solution for preliminary clinical scar assessment. To further advance the proposed framework, several directions warrant future investigation. First, integrating anatomical localization and scar segmentation into a unified end-to-end framework could effectively suppress inter-stage error propagation. Second, transitioning from the current slice-by-slice paradigms to full volumetric modeling is expected to enhance inter-slice spatial coherence. Future work will also focus on integrating robust prior knowledge and refining pseudo-label learning strategies to mitigate the limitations of weak supervision. Finally, a systematic evaluation of computational efficiency and model complexity, coupled with rigorous validation on larger, multi-center datasets and benchmarking against state-of-the-art transformer-based or hybrid architectures, will be essential to fully establish the clinical utility of the proposed framework.

## 6. Conclusions

In this paper, we introduced a scribble-supervised framework for myocardial scar segmentation from LGE CMR images using scribble annotations. The framework first extracts the myocardial region of interest through a weakly supervised anatomical segmentation network, and then performs scar segmentation within the cropped ROI under a teacher–student learning scheme. To address the difficulty of incomplete scar recovery under sparse supervision, we introduce a local distance prior loss to guide local supervision propagation, a myocardium-constrained structural prior consistency loss to improve structural coherence, and a detail-aware feature enhancement module to strengthen boundary delineation and fine-detail representation.

Experimental results on the MyoPS++ and EMIDEC datasets show that the proposed framework achieves superior performance compared with existing scribble-supervised methods and remains competitive with fully supervised approaches. These results demonstrate the effectiveness of combining anatomical constraints, local prior guidance, and consistency learning for weakly supervised myocardial scar segmentation. The proposed method provides an effective and annotation-efficient solution for myocardial scar analysis in LGE CMR images. While the segmentation performance under sparse scribble supervision remains lower than fully supervised approaches, the framework can still offer clinically useful preliminary scar localization, potentially reducing manual workload and facilitating downstream analysis.

## Figures and Tables

**Table 1:** Performance comparison of our method with other methods on the MyoPS++ dataset. Values are presented as mean ± standard deviation. Bold numbers indicate the best performance. Statistical significance was evaluated using the Wilcoxon signed-rank test, where * and ** indicate p<0.01 and p<0.001.

Methods	DSC ↑	HD95 (mm) ↓	ASD (mm) ↓
USTM	0.486±0.117 *	16.421±14.048 **	6.083±6.151 **
DMPLS	0.338±0.096 **	27.982±13.110 **	10.547±6.660 **
ScribFormer	0.398±0.102 **	19.511±13.906 **	7.343±5.824 **
**SSMSNet**	0.527±0.127	13.827±14.353	4.542±5.583
U-Net^sup^	0.539±0.142	12.142±15.102	4.389±7.782
UNet++^sup^	0.515±0.136	12.362±15.584	4.338±8.274
nn-UNet^sup^	0.548±0.143	9.427±12.642	4.015±9.035

Note that the superscript sup indicates supervised methods.

**Table 2:** Performance comparison of our method with other methods on the EMIDEC dataset. Values are presented as mean ± standard deviation. Bold numbers indicate the best performance. Statistical significance was evaluated using the Wilcoxon signed-rank test, where * and ** indicate p<0.01 and p<0.001.

Methods	DSC ↑	HD95 (mm) ↓	ASD (mm) ↓
USTM	0.365±0.158 **	35.392±22.731 **	14.728±18.062 **
DMPLS	0.234±0.112 **	35.571±20.289 **	14.721±16.321 **
ScribFormer	0.303±0.135 **	31.521±21.296 **	12.796±16.579 **
**SSMSNet**	0.492±0.176	11.247±16.506	4.809±9.846
U-Net^sup^	0.472±0.206	11.593±21.758	6.569±15.279
UNet++^sup^	0.395±0.202	15.434±19.247	8.043±16.697
nn-UNet^sup^	0.563±0.198	12.701±8.431	2.659±7.821

Note that the superscript sup indicates supervised methods.

**Table 3:** Sensitivity analysis of myocardial scar segmentation performance under perturbed anatomical priors.

Perturbation Type	Prior Error	DSC ↑	HD95 (mm) ↓	ASD (mm) ↓
Dilation	0.272±0.043	0.527±0.208	17.989±22.106	7.338±17.092
Dilation	0.614±0.084	0.526±0.206	17.319±22.009	7.654±17.959
Dilation	1.026±0.126	0.501±0.204	21.120±22.709	8.381±18.464
Erosion	0.301±0.051	0.512±0.208	19.291±21.218	7.894±17.386
Erosion	0.738±0.114	0.502±0.209	18.612±21.975	8.009±15.959
Erosion	1.490±0.356	0.487±0.206	23.150±22.147	9.135±17.149

**Table 4:** Ablation experimental results for the range coefficient λ in the local distance prior.

λ	DSC ↑	HD95 (mm) ↓	ASD (mm) ↓
$\lambda^{5}$	0.506±0.143	16.489±14.071	6.261±7.645
$\lambda^{6}$	0.514±0.138	15.986±13.394	5.476±8.324
$\lambda^{7}$	0.527±0.131	15.286±13.512	5.162±6.742
$\lambda^{8}$	0.521±0.132	15.402±13.795	5.458±6.210

Note that $\lambda^{i}$ represents $e^{i}$.

**Table 5:** Ablation experimental results for the distance threshold $\tau_{d}$ in the local distance prior loss function.

Setting	DSC ↑	HD95 (mm) ↓	ASD (mm) ↓
0.70	0.521±0.205	20.867±22.041	8.282±17.647
0.75	0.509±0.218	18.561±21.795	8.326±17.126
0.80	0.503±0.211	23.452±21.293	8.890±16.704
0.85	0.484±0.206	21.005±21.870	9.237±17.172
0.90	0.528±0.203	22.458±22.582	8.604±18.201

**Table 6:** Ablation experimental results of the confidence threshold $\tau_{p}$ in the structural prior consistency loss.

Setting	DSC ↑	HD95 (mm) ↓	ASD (mm) ↓
0.70	0.518±0.206	16.931±23.004	7.894±18.210
0.75	0.513±0.202	17.197±20.819	7.536±17.073
0.80	0.529±0.205	15.574±21.742	7.525±17.049
0.85	0.524±0.211	19.813±21.967	8.312±19.132
0.90	0.510±0.209	18.840±21.831	7.637±17.741
0.95	0.516±0.205	17.928±22.324	8.189±19.557

**Table 7:** Ablation experiment results for the contribution of each component and loss term in the proposed framework. TS indicates the teacher–student learning framework.

Methods	$L_{d}$	$L_{s}$	DFE	TS	DSC ↑	HD95 (mm) ↓	ASD (mm) ↓
#0	✗	✗	✗	✗	0.303±0.135	31.527±21.296	12.598±16.579
#1	✓	✗	✗	✗	0.331±0.143	26.136±22.180	11.949±16.627
#2	✗	✗	✓	✗	0.322±0.142	40.271±27.882	14.374±14.832
#3	✓	✗	✓	✗	0.352±0.168	28.988±21.808	12.843±16.652
#4	✓	✗	✗	✓	0.497±0.134	23.452±14.412	9.526±7.660
#5	✗	✓	✗	✓	0.401±0.186	25.946±22.200	11.174±17.487
#6	✗	✗	✓	✓	0.354±0.163	28.304±23.025	12.161±16.736
#7	✓	✗	✓	✓	0.508±0.151	21.645±13.182	10.450±11.093
#8	✗	✓	✓	✓	0.427±0.194	23.214±23.223	9.839±17.716
#9	✓	✓	✗	✓	0.516±0.131	18.624±13.974	6.982±7.018
#10	✓	✓	✓	✓	0.527±0.131	13.828±13.516	4.546±4.713

## Data Availability

To make our annotated dataset available to the research community for further study, it has been uploaded to https://github.com/Aven0261/SSMSNet (accessed on 18 May 2026).
